# Lateral intraparietal area (LIP) is largely effector-specific in free-choice decisions

**DOI:** 10.1038/s41598-018-26366-9

**Published:** 2018-06-05

**Authors:** Vassilios N. Christopoulos, Igor Kagan, Richard A. Andersen

**Affiliations:** 1 Division of Biology and Biological Engineering, California Institute of Technology, Pasadena, CA USA; 2 Decision and Awareness Group, Cognitive Neuroscience Laboratory, German Primate Center, Leibniz Institute for Primate Research, Goettingen, Germany; 3 The Tianqiao and Chrissy Chen Brain-Machine Interface Center, California Institute of Technology, Pasadena, CA USA; 4 Leibniz Science Campus Primate Cognition, Goettingen, Germany

## Abstract

Despite many years of intense research, there is no strong consensus about the role of the lateral intraparietal area (LIP) in decision making. One view of LIP function is that it guides spatial attention, providing a “saliency map” of the external world. If this were the case, it would contribute to target selection regardless of which action would be performed to implement the choice. On the other hand, LIP inactivation has been shown to influence spatial selection and oculomotor metrics in free-choice decisions, which are made using eye movements, arguing that it contributes to saccade decisions. To dissociate between a more general attention role and a more effector specific saccade role, we reversibly inactivated LIP while non-human primates freely selected between two targets, presented in the two hemifields, with either saccades or reaches. Unilateral LIP inactivation induced a strong choice bias to ipsilesional targets when decisions were made with saccades. Interestingly, the inactivation also caused a reduction of contralesional choices when decisions were made with reaches, albeit the effect was less pronounced. These findings suggest that LIP is part of a network for making oculomotor decisions and is largely effector-specific in free-choice decisions.

## Introduction

Over the past several years, a growing body of studies provided evidence that the lateral intraparietal area (LIP), a subdivision of the inferior parietal lobule (IPL), is involved in various computations related to decision making^1,2^. The evidence is mainly based on neurophysiological recordings showing that LIP neurons integrate many factors related to choices^2,3^, represent decision-related variables, such as expected reward and outcome probability^4–8^, and accumulate sensory evidence over time for making perceptual judgments^9,10^. Along the same lines, pharmacological inactivation studies showed that temporary lesions in LIP caused a reduction of contralesional choices – i.e., the visual space represented by the silenced neurons – in oculomotor free-choice decisions^11,12^. Remarkably, silencing LIP neurons in motion discrimination tasks did not have any effect on the decision process^13^, questioning the role of LIP in perceptual decisions^14,15^. Similar findings have also been reported in rodents, where inactivating PPC neurons whose activity was correlated with decision-related variables affected free-choices but not perceptual judgments^16^.

While these studies revealed a key role of LIP in oculomotor free-choices, it is still unclear whether it contributes to decision-making irrespective of which action is performed to implement the selected option (i.e., effector non-specific hypothesis) or it is involved mainly in oculomotor decisions (i.e., effector-specific hypothesis). An effector non-specific deficit after inactivation would be consistent with the attention role for LIP in decision making, in which LIP guides the allocation of spatial attention to select a target in space, regardless on how this target will be used^17–21^. To dissociate between a more general attention role and a more effector-specific saccade role, we reversibly inactivated LIP while two macaque monkeys performed memory-guided saccade or reach movements to either a single target or selected one of two targets presented simultaneously in both hemifields. Consistent with previous studies^11,12,22,23^, after silencing LIP neurons the animals were less likely to select saccade targets located in the contralesional hemifield. At the same, LIP inactivation caused a reduction of contralesional reach choices, albeit the effect was less pronounced. The sensory, memory and motor components of the task remained largely intact, besides a reduction of the reach performance in one animal for movements to both visual hemifields. Overall, our results provide direct evidence that LIP is primarily saccade-specific in free-choice decisions, although it likely encodes also some components of more global processes, unlike the parietal reach region (PRR), which is nearly entirely reach-specific^24^.

## Results

**Figure 1:**
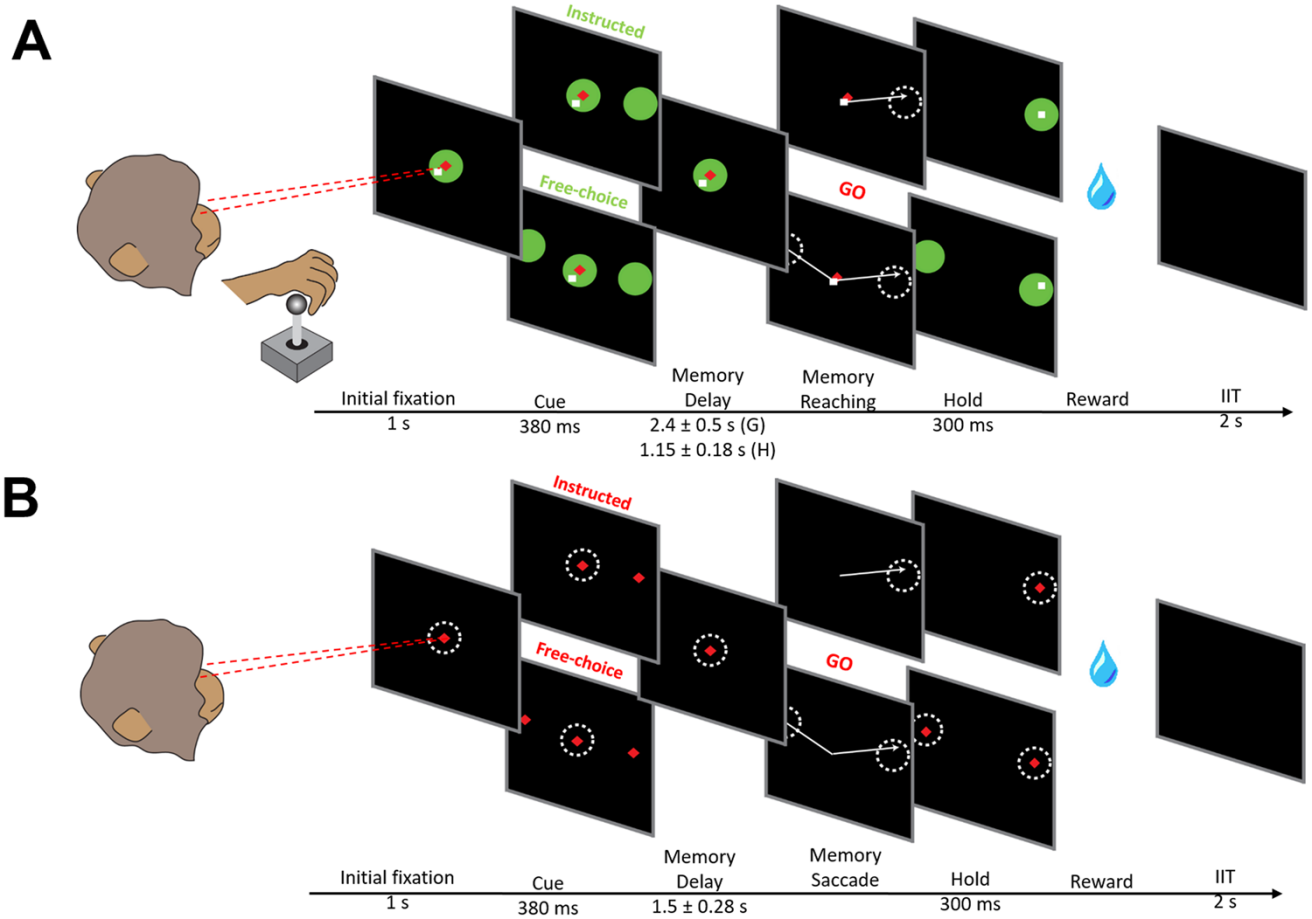
(**A**) Memory-guided reaching task. The animals sat in an upright position in a primate chair at a distance approximately 30 cm from the screen. A 2-dimensional joystick was positioned in front of the sitting animals, with the handle at the level of their knees. A trial started with two central fixation cues, which the animals had to acquire with their eyes (red diamond) and with the joystick (green circle). Next, either a single green cue was presented in the left or the right hemifield (instructed trials) or two green cues were presented simultaneously in both hemifields (free-choice trials). The cues(s) disappeared after 380 ms; the animals had to remember their location during the delay period, and could select and plan the upcoming movement. After the green central fixation cue was extinguished (go signal), the animals had to make a reaching movement to the instructed or chosen target and wait there for another 300 ms to receive the reward. Importantly, the animals had to maintain eye fixation on the central red diamond cue throughout the reaching trial. (**B**) Memory-guided saccade task. It was similar to the reaching task described in A with the difference being that choices were made using eye movements (saccades). Red diamond cues indicate the location of the central fixation and the saccade targets.

**Figure 2:**
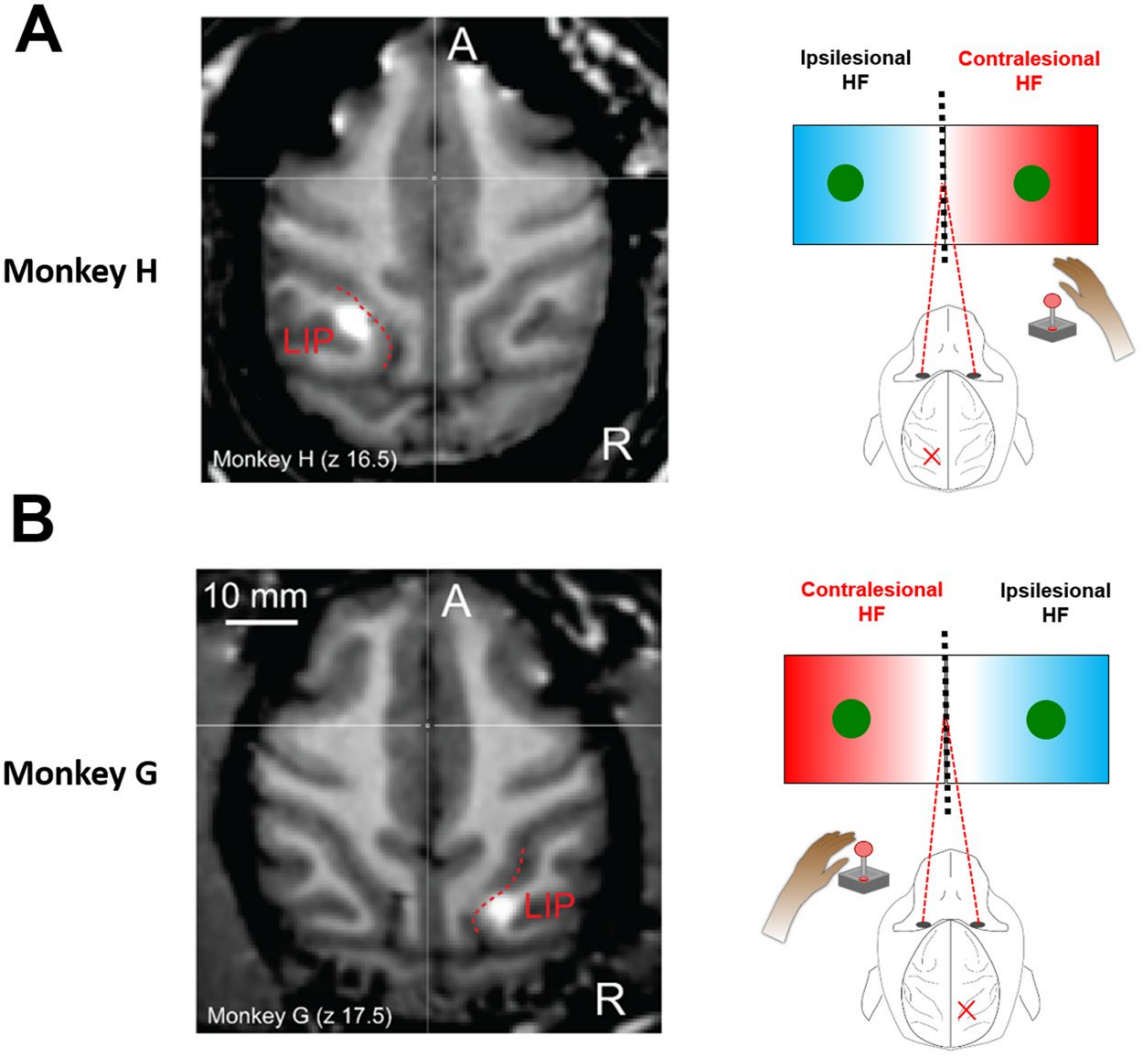
(**A**) *Left*: Horizontal MR section visualizing the injection site for monkey H with gadolinium MR contrast agent (white). The MR images were acquired 30–45 min after a 5.5 μl infusion of gadolinium. *Right*: Drug injections were performed in the left LIP, contralaterally to the right hand this animal used to control the joystick. Hence, the left and the right hemifields are the ipsilesional and the contralesional sides, respectively, for monkey H. (**B**) Similar to A, but for monkey G (5.5 μl infusion of gadolinium). This monkey was using the left hand to control the joystick, thus the drug injections were performed in the right LIP. The left and the right hemifields are the contralesional and ipsilesional sides for monkey G. In both monkeys, the extent of the gadolinium spread in the coronal sections (not shown here) indicated the dorsal part of the LIP (LIPd) was primarily affected (in monkey H, also a small portion of the adjacent LIPv was affected).

We explored the effects of the LIP inactivation on free-choice decisions by local injection of muscimol while two monkeys performed memory-guided reach (Fig. 1A) and saccade (Fig. 1B) movements to either a single or one of two targets presented simultaneously in both hemifields. Inactivation sites were localized by injecting the MRI-visible contrast agent gadolinium, which is known to correspond closely to the spread of muscimol^25^, and subsequently imaging its spread using a 3T horizontal bore scanner (Fig. 2). The spread of gadolinium confirmed that injections were performed within a restricted volume of the lateral bank of the intraparietal sulcus, corresponding to LIP, primarily the dorsal aspect (LIPd, Fig. 2A,B for monkeys H and G, respectively).

### Inactivation effects in instructed trials

**Figure 3:**
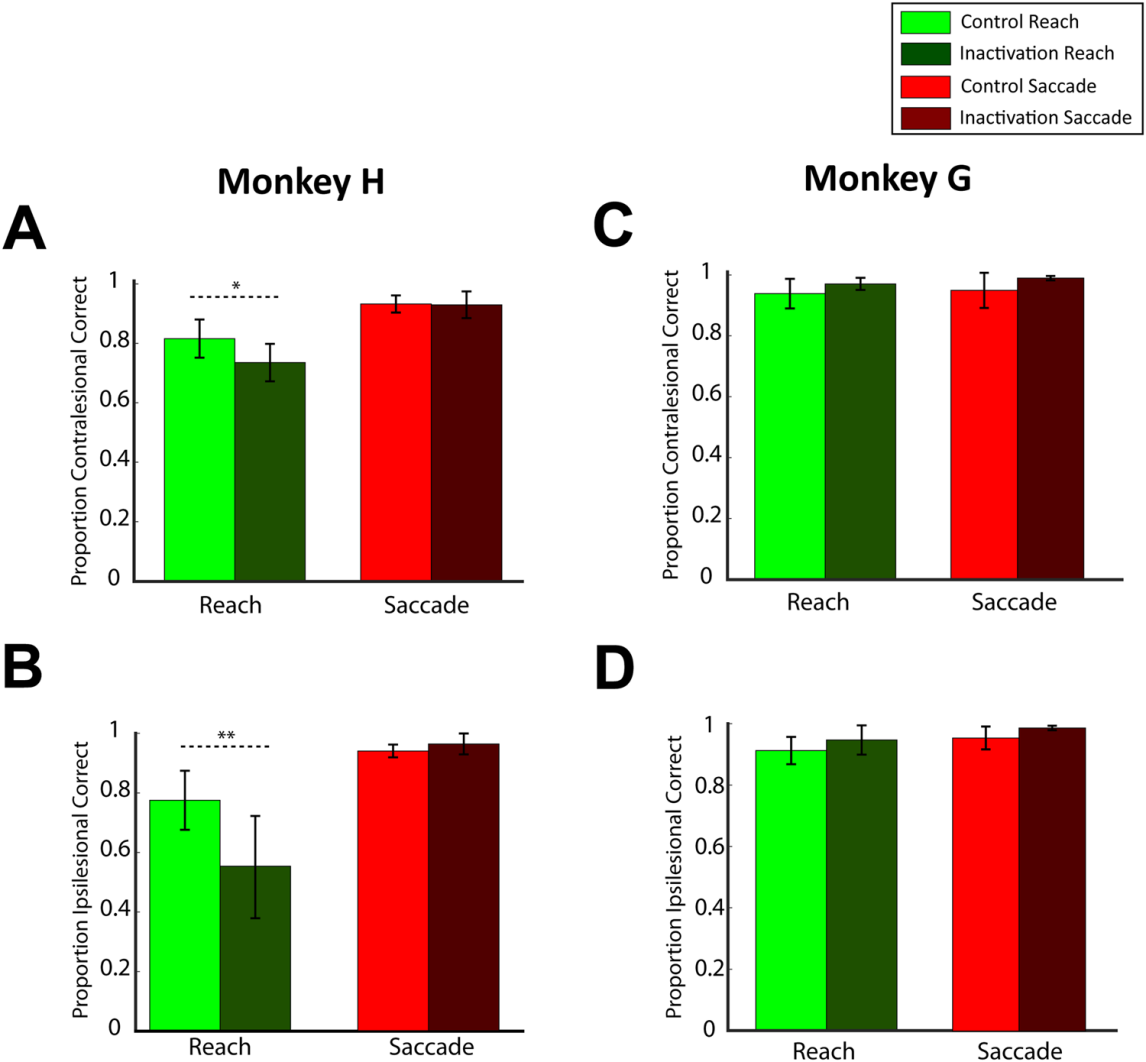
Proportion of correct saccades and reaches to (**A**–**C**) contralesional and (**B**–**D**) and ipsilesional sides for monkey H (left column) and monkey G (right column) during control (light green and light red for reaches and saccades, respectively) and inactivation (dark green and dark red for reaches and saccades) sessions. The error bars correspond to SD across all sessions (13 control and 9 inactivation sessions for monkey H, and 11 control and 4 inactivation sessions for monkey G). Drug injection reduces the reach performance for movements to each hemifield for monkey H (two-tailed *t* test analysis, p = 0.0085 for contralesional and p = 0.00092 for ipsilesional reaches), but not for monkey G. No significant changes were found on saccade performance (two-tailed *t* test analysis, p > 0.05). ^*^p < 0.05, ^**^p < 0.001.

**Figure 4:**
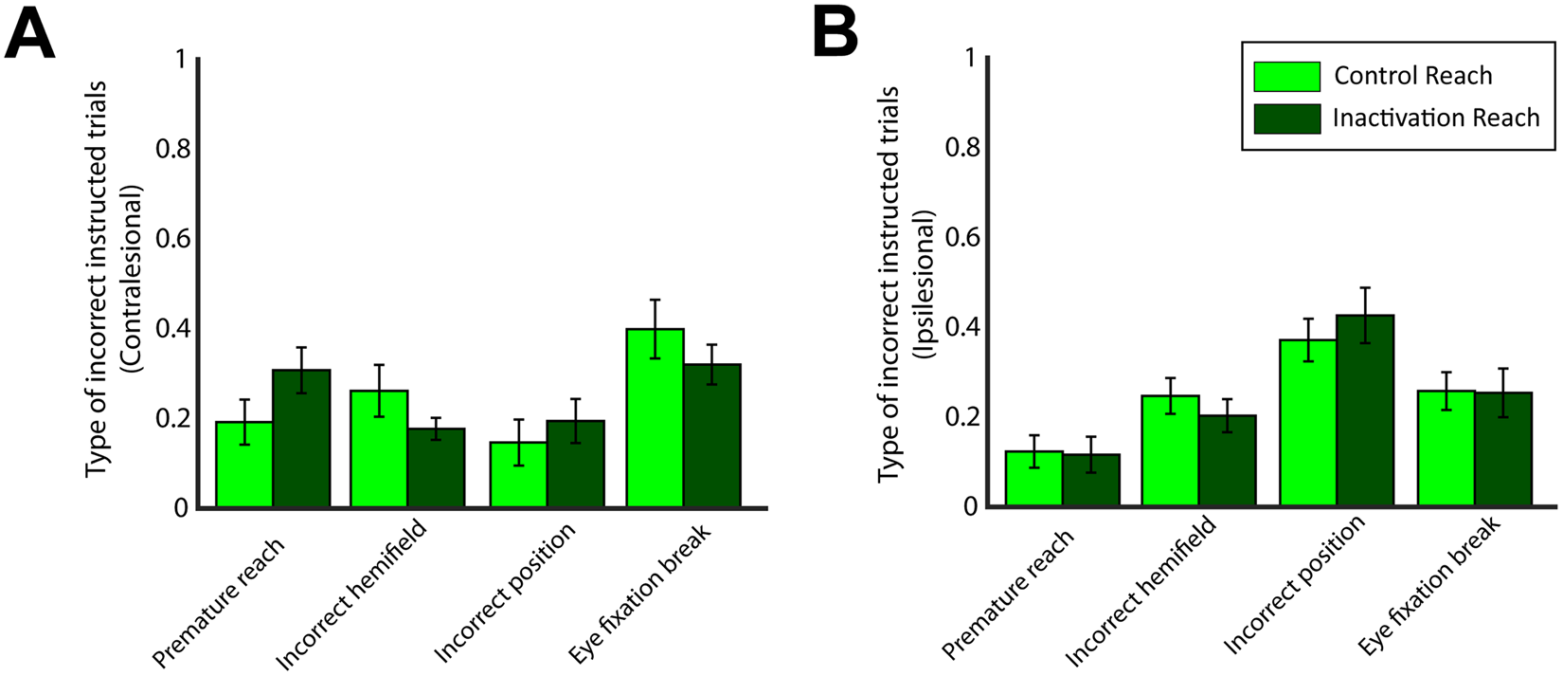
We defined the following types of errors in the reach instructed trials for monkey H: (i) premature reach movement (error type 1); (ii) reach movement to incorrect hemifield (error type 2); (iii) reach movement to incorrect location within the correct hemifield (error type 3) and (iv) eye fixation break at any moment during the trial (error type 4). Panel (**A**) illustrates the relative proportion of the error types for reaches to the contralesional hemifield in control (light green) and inactivation (dark green) sessions, normalized to 100% independently for control and inactivation sessions. This normalization approach was adopted to assess a potential relative increase or decrease of specific error types after the inactivation. (**B**) Similar to panel A, but for ipsilesional trials. Mixed factorial ANOVAs within each error type (factors treatment × hemifield) did not reveal any significant differences between control and inactivation sessions, nor the interaction between treatment and hemifield. There was a main effect of hemifield (F(1,20) > 8, p < 0.004) for all types of errors, except for the error type 2 (i.e., reaches to incorrect hemifield), indicating that while the relative distribution of different error types differed in the two hemifields, these differences were same in control and inactivation sessions.

We examined the consequences of LIP inactivation on the instructed trials to test whether the drug injection affected the sensory, memory and motor components of the saccade and reach tasks. We first evaluated the performance of the animals in the control and inactivation sessions by computing the proportion of correctly executed reach and saccade trials. Figure 3 depicts the performance of monkeys H and G for reaches and saccades made to the contralesional (panels A and C) and the ipsilesional (panels B and D) side of space. We found that the drug injection affected the reach performance in monkey H, who already had a lower performance for reaches than for saccades in the control sessions. A mixed ANOVA (factors treatment × effector, treatment between sessions, effector within sessions, see Methods) analysis indicated that in addition to a main effect of effector (F(1,20) > 105, p < 10^−7^ for both sides of space) there was a main effect of treatment in ipsilesional (F(1,20) = 10.8, p = 0.0036) and contralesional (F(1,20) = 6.2, p = 0.021) instructed trials. There was also an interaction between effector and treatment in ipsilesional (F(1,20) = 18.9, p = 0.0003) and contralesional (F(1,20) = 6.7, p = 0.017) reaching movements. A two-tailed *t* test analysis across control and inactivation sessions, performed separately for each hemifield, showed that the performance was substantially reduced for both contralesional and ipsilesional reach movements (p = 0.0085 for contralesional and p = 0.00092 for ipsilesional reaches), but not for eye movements (p > 0.05). Note that the effect was stronger for reaches to the ipsilesional hemifield (performance reduction from 77% to 55%, 22%) rather than to the contralesional hemifield (reduction from 81% to 73%, 8%), as also indicated by the interaction between the factors *hemifield* (i.e., left vs. right) and *effector* in a separate mixed ANOVA analysis (factors treatment × hemifield, treatment between sessions, hemifield within sessions) across the reach sessions (*treatment*: F(1,20) = 16.4, p = 0.0006; *hemifield*: F(1,20) = 19.9, p = 0.0002; *interaction*: F(1,20) = 8.085, p = 0.01). To further assess the effects of drug injection on the reaching performance of monkey H, we evaluated four types of errors in the instructed reach trials: (1) premature reach (reach initiation prior to go signal), (2) incorrect hemifield (reach after the go signal but to the opposite hemifield from where the target is located), (3) incorrect position (reach after the go signal to  an incorrect location within the correct hemifield) and (4) eye fixation break (eyes move out the tolerance window at any moment during the trial). Figure 4A,B illustrates the relative proportion of these error types within the incorrect contralesional and ipsilesional trials, respectively, before and after LIP inactivation. We performed a mixed ANOVA analysis across the reach sessions separately for each type of error, with *hemifield* (i.e., left vs. right movement) as a within-subject factor and *treatment* (control vs. inactivation) as a between-subject factor. We found no main effect of treatment in any type of errors (F(1,20) < 1.5, p > 0.2) suggesting that drug injection did not disproportionally influence any specific error type in the reaching movements. Also, no significant effect on the interaction between treatment and hemifield was found for all types of errors (F(1,20) < 2.4, p > 0.1).

In the other animal (monkey G), who had a high performance for both effectors in the control sessions, a mixed ANOVA revealed a slight but statistically significant performance improvement on both reaches and saccades, in both hemifields after the inactivation (main effect of treatment, F(1,13) > 5, p < 0.05). Since the performance of the animal G was very high, we did not perform error type analysis as we did for monkey H. Overall, LIP inactivation left memory saccades to instructed single targets intact^12^, while it decreased the proportion of correctly completed instructed reaches to both hemifields in one of the animals.

**Figure 5:**
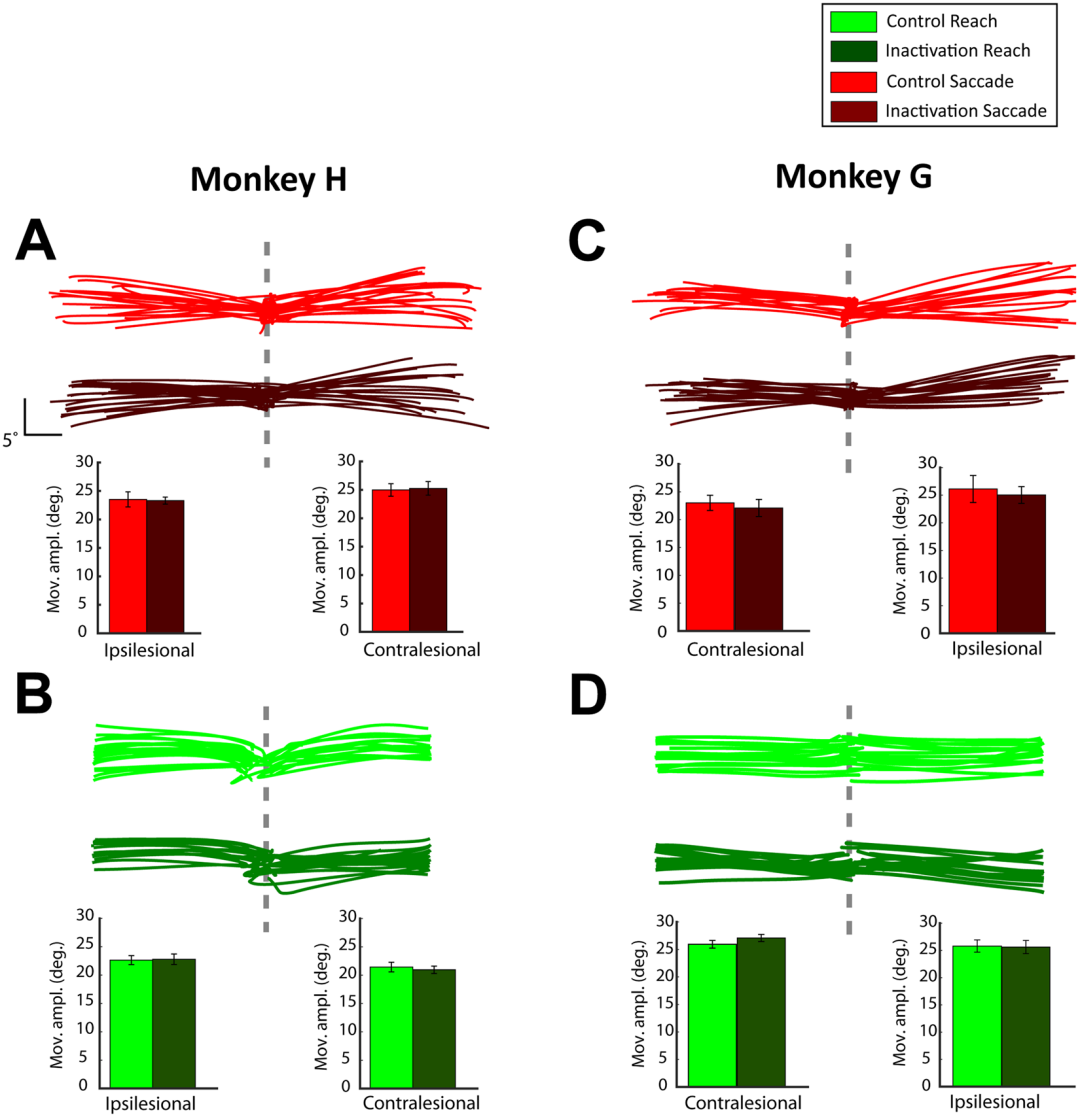
(**A**) *Top panel*: Saccade trajectories in instructed trials from representative control (light red) and inactivation (dark red) sessions for monkey H. *Bottom panel*: Average movement amplitude for instructed saccades to ipsilesional (left) and contralesional (right) targets, across all control and inactivation sessions. (**B**) Similar to A, but for control (light green) and inactivation (dark green) reach trials. Panels (**C**) and (**D**) are similar to panels A and B, respectively, but for monkey G. The error bars correspond to SD across all sessions (13 control and 9 inactivation sessions for monkey H, and 11 control and 4 inactivation sessions for monkey G). A mixed factorial ANOVA did not reveal any significant differences on the movement amplitude between control and inactivation sessions in both reach and saccade instructed trials. There was a main effect of effector in both animals (contralesional: monkey H: F(1,20) = 163.303, p < 10^−6^, monkey G: F(1,13) = 68.521, p < 10^−6^; ipsilesional: monkey H (F(1,20) = 4.978, p = 0.037, but not in monkey G (F(1,13) = 0.04, p = 0.845), but this was likely due to slightly different gains for the calibration of eye and joystick movements, especially in monkey H (note smaller amplitudes of joystick trajectories as compared to saccades, both in control and inactivation sessions).

We also tested whether drug injection affected the reach and/or saccade amplitude by inducing hypometric movements (i.e., optic ataxia (OA)-like symptoms^26,27^). Top panels in Fig. 5A–D illustrate saccade and reach trajectories from representative control and inactivation sessions for both animals. The trajectories in the control sessions were not noticeably different from the trajectories in the inactivation sessions in both reach and saccade single-target trials. We quantified the effects of muscimol injections on the movement amplitude by comparing the distance between the origin and the endpoint of the movements in the control and inactivation sessions. The bottom panels in Fig. 5A–D depict the average saccade and reach amplitude across all control versus inactivation sessions for the contralesional and ipsilesional targets. We found no significant main effect on the movement amplitude after the drug injection for both reach and saccade trials, as indicated by the lack of main effect due to treatment factor in the mixed ANOVA analysis (monkey H: F(1,20) < 0.105, p > 0.7, monkey G: F(1,13) < 0.7 and p > 0.4 for instructed trials in both hemifields). Importantly, no statistically significant effects were found on the interaction between the treatment and effector (monkey H: F(1,20) < 1.9, p > 0.128, monkey G: F(1,13) < 4.5 and p > 0.05). Additionally, LIP inactivation did not affect the movement latency for reaches and saccades to contralesional targets. Although the lack of effect on saccade latency has been previously reported in oculomotor tasks^23^, other studies have  shown that monkeys exhibit a modest increase of saccade latencies (5 to 16 ms) towards contralesional targets after LIP inactivation^12,28,29^. While it is likely that drug injection did not affect the movement latency in our study, it is important to note that the lack of effect may be related to other factors, such as the low sampling rate of the eye and joystick cursor recordings (62.5 Hz). Previous studies that reported modest changes of the saccade latency after silencing the LIP neurons acquired eye movements with a faster sampling rate. Differences in the design of the experiment can also explain why we found no effects of drug injection on saccade and reach latency. In particular, inactivation and control trials were not performed within the same sessions. Instead, they were spaced by at least 24 hours apart. Movement latency is sensitive to parameters such as the motivation of the animals, which can vary across days and within the session with satiety/tiredness. Overall, our findings suggest that the sensory, memory, oculomotor and reaching capabilities of the animals remained largely intact after LIP inactivation.

### Inactivation effects in free-choice trials

**Figure 6:**
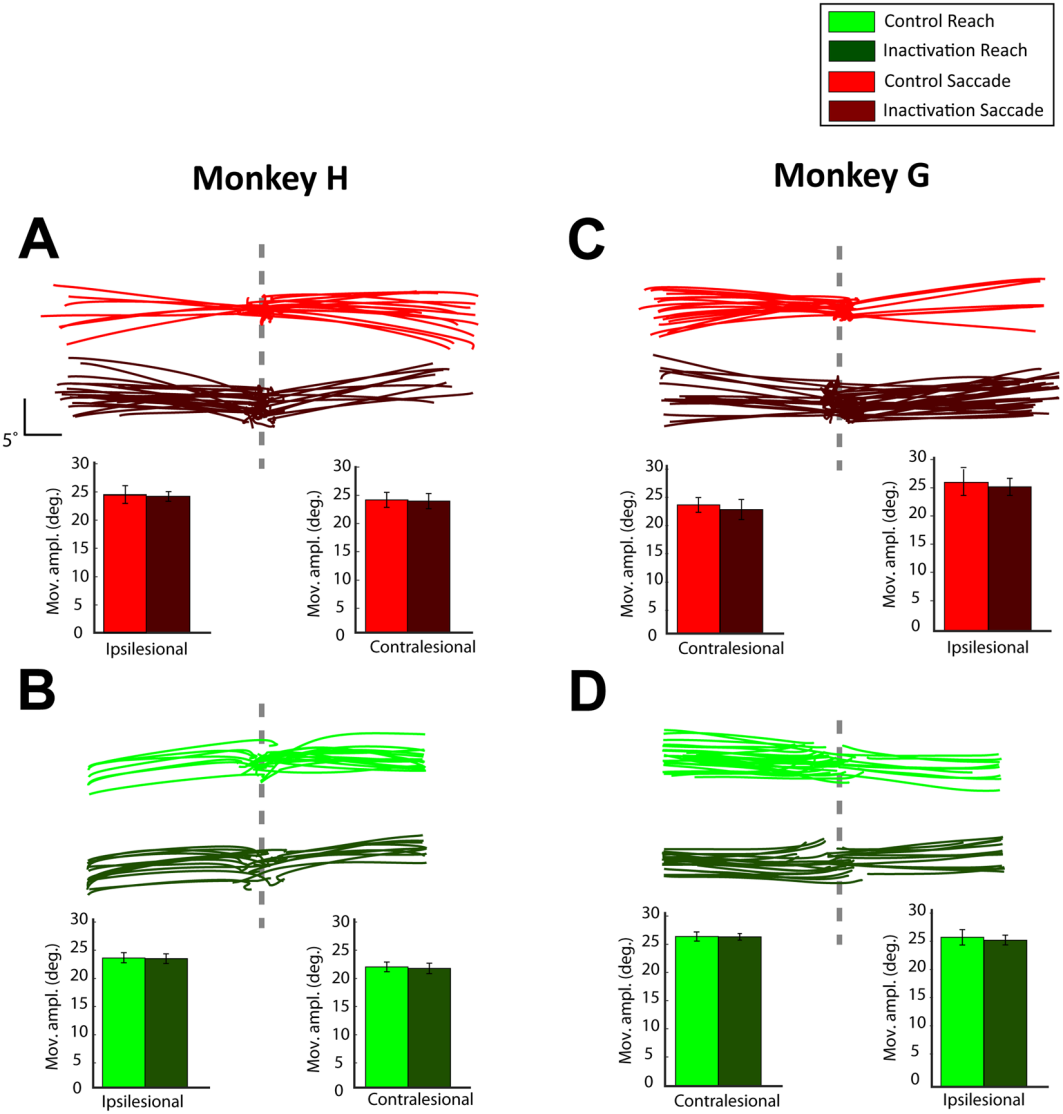
Similar to Fig. 5, but for the free-choice trials. A mixed factorial ANOVA did not reveal any significant differences in the movement amplitude between control and inactivation sessions in both reach and saccade free-choice trials.

Similar to the single-target instructed trials, we also tested whether LIP inactivation affected the amplitude of the reach and saccade movements in the two-target free-choice trials. Figure 6A–D illustrate reach and saccade trajectories from representative control and inactivation sessions for both monkeys H and G. Consistent with the findings in the instructed trials, there were no noticeable differences on the movement amplitude in both reach and saccade movements after LIP inactivation. We quantified the effect of LIP inactivation on the movement amplitude (bottom panels in Fig. 6A–D) by performing mixed ANOVA analysis (factors treatment × effector) and found no effect of treatment for both contralesional and ipsilesional amplitudes in both animals (monkey H: F(1,20) < 0.5, p > 0.5, monkey G: F(1,13) < 0.98, p > 0.3 for free-choice trials in both hemifields). No statistically significant effects were found on the interaction between the treatment and effector in both animals (monkey H: F(1,20) < 0.07, p > 0.8, monkey G: F(1,13) < 0.7, p > 0.4). Also similarly to the instructed trials, we found no effects of drug injection on the reach and saccade latencies.

**Figure 7:**
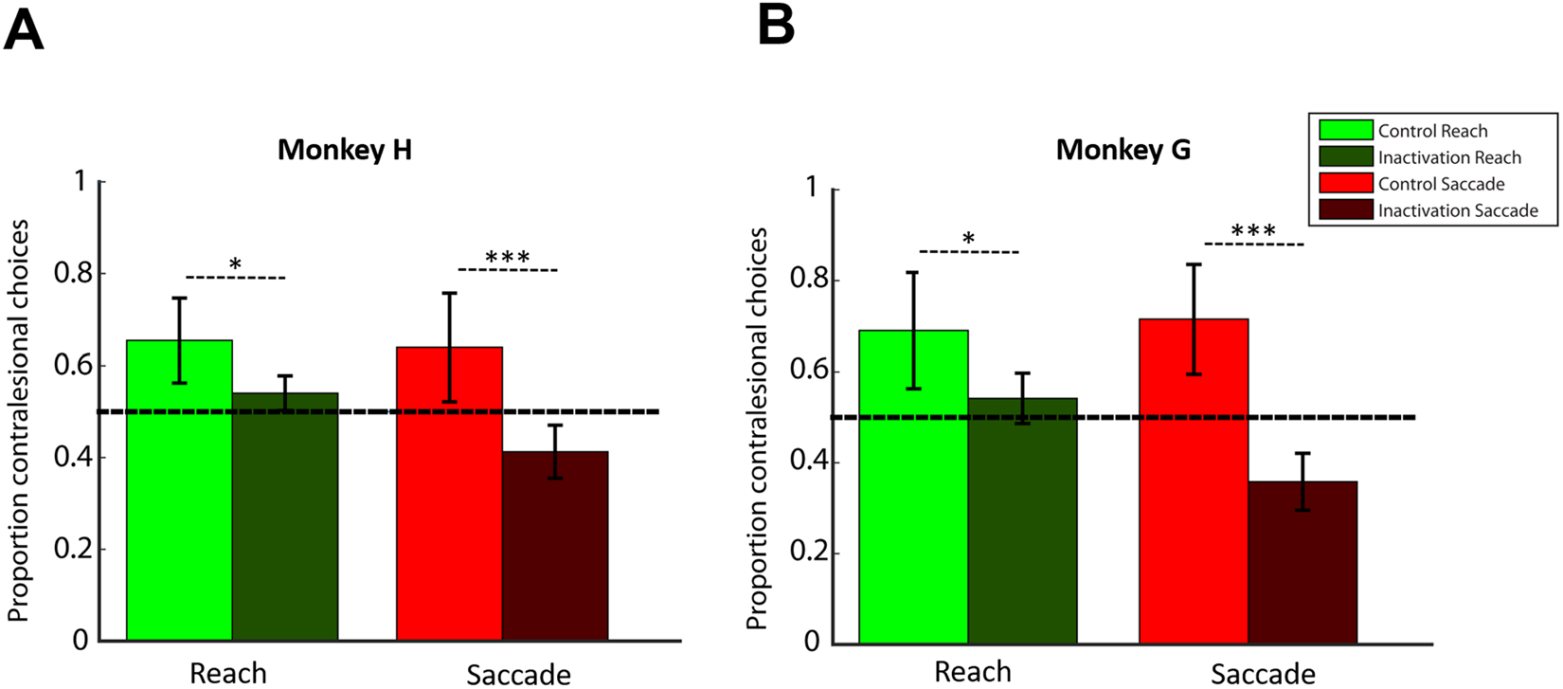
(**A**) Proportion of contralesional reach and saccade choices for monkey H across control and inactivation sessions. The error bars correspond to SD across sessions (13 control and 9 inactivation sessions). (**B**) Similar to A, but for monkey G (11 control and 4 inactivation sessions). LIP inactivation caused a reduction of contralesional saccade and reach choices in both animals. However, the effect was stronger when choices were made using eye rather than hand movements, as indicated by the interaction between the factors *treatment* and *effector* (mixed factorial ANOVA main effect on *treatment* for contralesional choices in monkey H: F(1,20) = 39.405, p = 4 × 10^−6^ and monkey G: F(1,13) = 27.277, p = 1.64 × 10^−4^, main effect on *interaction* in monkey H: F(1,20) = 4.487, p = 0.04 and in monkey G: F(1,13) = 5.511, p = 0.035). ^*^p < 0.05, ^***^p < 0.0001 from a separate two-tailed *t* test analysis.

Next, we evaluated the effects of the drug injection on choices between the two equally rewarded targets to test whether LIP exhibits effector specificity for free-choice decisions. Although individual spatial preferences were apparent in the control sessions (both monkeys happened to more often select the targets contralateral to the injection hemisphere in both saccade and reaching tasks, perhaps due to less effort in moving to the side of space congruent with the acting hand), the animals chose both sides of space in the free-choice trials, Fig. 7A,B. We evaluated the effects of drug injection on target selection and found that LIP inactivation significantly reduced the contralesional reach and saccade choices (mixed ANOVA, main effect of *treatment* on contralesional choices in monkey H: F(1,20) = 39.405, p = 4 × 10^−6^ and in monkey G: F(1,13) = 27.277, p = 1.64 × 10^−4^). Importantly, the effect was stronger when choices were made using eye (23–36% reduction) rather than hand (11–12% reduction) movements, as also indicated by the interaction between the factors *treatment* and *effector* (mixed ANOVA, *interaction* effect in monkey H: F(1,20) = 4.487, p = 0.04 and in monkey G: F(1,13) = 5.511, p = 0.035). A post hoc two-tailed *t* test analysis across sessions revealed a significant reduction of contralesional choices for saccades (p < 0.0001) and reaches (p < 0.05) in both animals. Note that although the LIP inactivation did cause a weak reduction of contralesional reach choices, the choice bias did not flip to a preference for targets on the ipsilesional side, as occurred in the free-choice saccade trials. Overall, our findings suggest that LIP plays an important role in oculomotor decisions, and is largely but not entirely saccade-specific.

## Discussion

Decision making has been traditionally considered a centralized cognitive process, which resides in the frontal lobes and is separate from the neural systems for perception and action^30–33^. However, recent evidence suggests that decisions between physical actions also involve cortical regions implicated in planning and generating actions^34–41^. Part of the evidence comes from reversible pharmacological inactivation studies in monkeys^12,22–24,42^ and rodents^16^ showing that silencing neurons in the  posterior parietal cortex and in the pulvinar biases free-choices towards the contralesional visual field. A recent study from our lab went a step further and showed that silencing PRR neurons, which are involved in planning of reaches, affects only free-choice decisions that are made using hand movements^24^. Here, we explored whether such an effector-specificity in decision making holds also for area LIP.

To address this question, we reversibly inactivated a portion of LIP by injecting the GABA-A agonist muscimol, while two animals performed memory-guided reach and saccade choices to two targets presented simultaneously in both hemifields. Both targets provided the same amount of reward and the animals were free to choose either of them after a short memory delay. Free-choice trials were interleaved with instructed trials, in which a single target was presented either in the left or the right visual field. Besides a reduction of reach performance for movements to both hemifields in one of the two animals, silencing the LIP neurons left largely intact the sensory, memory and motor components of the saccade and reach task performance. However, the inactivation caused a pronounced bias towards the ipsilesional hemifield for saccade choices as has been already reported in previous studies^11,12,22^. Interestingly, LIP inactivation also influenced the reach choices, but the effect was less pronounced than in the saccade task. Both animals exhibited a slight, but statistically significant, reduction of contralesional reach choices, without shifting their overall choice preference to targets located in the ipsilesional hemifield. These results suggest that LIP has a key role in oculomotor decisions, although it may not be fully effector-specific such as reach-specific PRR.

The effects of muscimol injection on reach choices could suggest that LIP predominantly supports functions such as attention^43–45^ and visual categorization (i.e., group of left targets vs. group of right targets)^46,47^ that are only spatial- and not effector-specific. However, this hypothesis is not supported by the different size of the effect on the reach and saccade choices. If LIP represented only spatial global information, muscimol injection would produce similar choice bias in both reach and saccade choices.

An alternative explanation that could account for the reach choice bias is the strong interconnection between the PPC areas that are involved in reach and saccade movements. Reach and eye movement areas must interact to coordinate complex behavior such as eye-hand coordination^48^. In particular, inactivation studies in monkeys have shown that silencing LIP^28^ and PRR^49^ neurons affects both reaches and saccades in joint eye-hand coordination movements. Along the same lines, perturbing the dorsal pulvinar, the thalamic structure with strong connections to both LIP and PRR, affects both saccade and reach choices^42,50,51^. Additionally, neurophysiological studies identified LIP neurons that are correlated with the reaction times of concurrently planned, coordinated hand and eye movements^52^. These neurons are coherent with the beta band local field potentials (LFPs), which are also correlated with the reaction time of eye and hand movements in both LIP and PRR^52,53^. These findings suggest that LIP and PRR are heavily interconnected and exchange information about eye and hand movements, and therefore inactivating LIP could also influence reach choices. Additionally, a recent study reported that LIP neurons represent the relative desirability (i.e., how desirable it is to select one option over the alternatives) for both reach and saccade choices in value-based decisions^6^. Importantly, the representation was at least twice as strong when choices were made using eye rather than arm movements, suggesting that silencing LIP neurons should have stronger effects on saccade than reach choices, in agreement with our results. On the other hand, PRR neurons encode target desirability only when decisions were made using hand movements and therefore silencing PRR neurons should bias only reach choices, in agreement with our previous study^24^.

The complementary, although not completely mirror-symmetrical, roles of LIP and PRR in free-choices with saccades and reaches are further supported by a recent study that investigated summation patterns of information concerning the spatial location and the effector for the movement selection^54^. In this study, PRR exhibited a stronger effector-specificity than LIP, especially during the movement, but in agreement with previous studies, LIP also showed effector-specificity during movement planning, leading Chang *et al*. to favor the interpretation that LIP is saccade-specific (but not sufficient for saccade generation). Thus, when the effector is specified, the LIP inactivation affects resolving the competition between the saccade targets more than between the reach targets, but since 1) LIP projects to areas that encode reaches and 2) because in natural behavior reaches are very frequently preceded by saccades, the weak effect on reaches is present as well. The non-effector-specific component might also be related to the exogenous bottom-up salience of spatial locations and top-down endogenous attentional modulation from FEF to LIP^55^.

Our findings are partially at odds with the results from a recent study by Kubanek *et al*. reporting that LIP inactivation biases only saccade choices, whereas hand choices remain intact^22^. The differences in the experimental procedures of the two studies could account for the contradictory results. Kubanek *et al*. used the double-target paradigm within the context of a stimulus onset asynchrony (SOA) task with a variable delay between the onsets of the two targets. The animals were trained to report which target appeared earlier to receive reward with 60% probability. This is a perceptual decision-making task (i.e., temporal order judgment) rather than a free-choice task (free choice between targets associated with equal and deterministic reward) as used in our study. Neurophysiological recordings have provided evidence that LIP contributes differently to these two types of decisions. LIP neurons seem to have a key role in integrating sensory information during the formation of perceptual decisions^9,10,56^, although recent studies arguing against this hypothesis showed that silencing LIP neurons in monkeys^13^ and PPC neurons in rodents^16^ does not have any impact on the decision accuracy in motion direction discrimination tasks. These new findings suggest that LIP is not causally involved in perceptual judgments, and the decision-related activity in LIP may be related to feedback from other brain regions that contribute to the decision-making process^57^, or an emergent phenomenon driven by extensive training^58^. On the other hand, neurophysiological studies of value-based decisions reported that LIP neurons might integrate value information from disparate sources into a common currency to evaluate the alternative options^2,36,59,60^. The different role of LIP in perceptual and free-choice decisions, as well as other important differences between the two studies, such as natural arm reaches vs. reaches with a joystick, and immediate choices vs. memory-guided choices, may explain the different patterns of inactivation-induced deficits.

Finally, it could be argued that the different level of difficulty and attentional allocation in the two behavioral tasks might be a confounding factor in our findings. First, the memory-guided saccade and reach tasks do not match in terms of difficulty, since the animals had to maintain eye fixation while reaching to the peripheral cues in the reach trials. Indeed, animal H performed worse in the reach than in the saccade trials. However, if the differential effect of the LIP inactivation on the saccade and reach choice behavior was primarily driven by the task difficulty, we would expect more impairment, i.e. stronger reduction of contralesional choices, when decisions are made using joystick movements than eye movements, the opposite to what we found. Additionally, animal G, who achieved a high level and nearly equal performance in both saccade and reach tasks, exhibited the same effect on choice behavior as animal H after LIP inactivation. Furthermore, previous pharmacological inactivation studies that used a similar comparison of these tasks reported inactivation-induced patterns that are not consistent with a predominant role of the task difficulty. For instance, the study of Kubanek *et al*.^22^, discussed above, found that LIP inactivation affected only the perceptual saccade choices but not the reach choices that also required a central fixation (whereas PRR inactivation affected only the reach choices), suggesting that difficulty per se was not a strong contributing factor. To summarize, an argument can be made that the presence of a small but significant effect on reach choices after LIP inactivation in our study is driven by the increased difficulty of the reach task. Even if this were the case, it does not invalidate our main conclusion that LIP is at least partially saccade-specific. If anything, this would suggest that if the task difficulty were somehow equalized, the LIP inactivation would affect the reaches even less, and thus show more saccade specificity.

Another related concern is that the two tasks were different during the execution phase in terms of attentional allocation. It is indeed likely that during the reach movement, attention was divided between the peripheral target position and the foveal fixation loci, while in the saccade task, the attentional locus switched fully to the target location after the go signal. This difference reflects inherent ‘asymmetry’ between the eye movements, which are typically linked to attention (or vice versa) but often are dissociated from hand movements, and hand movements, which are typically (although not always) coupled to preceding eye movements. We minimized this difference by employing the memory delay paradigm: in both tasks, after the spatial cue, monkeys could decide on and prepare the movement in advance during the delay period. Note that during the delay period, covert spatial attention is distributed between foveal fixation and peripheral target loci in both tasks, and only after the go signal does  the task difference in attentional distribution emerges. Since the inactivation in this and in previous studies^12,13,24^ did not result in strong primary movement deficits (almost no effect on single target instructed trials), it can be argued that the main effect of the inactivation on the spatial choices takes place before the movement, i.e. when there is no difference in attentional allocation between the tasks. Nevertheless, we cannot rule out that LIP involvement in spatial attention, suggested by other studies^43–45^, might have contributed to the observed effects on reaches.

Taken together with our recent findings from the PRR inactivation study^24^, these results support the theory that decision making involving immediate physical actions is not a centralized process residing in the frontal lobes. Instead, action decisions evolve within the same circuits that plan and generate particular motor actions^34,35,37,61^. PRR is more reach-specific in contrast to LIP which is largely saccade-specific but also encodes additional global components operating at the level of action decisions.

## Methods

### Experimental procedures and animal training

Two adult male rhesus macaques (*Macaca mulatta*) weighing 10–12 kg were implanted under general anesthesia with a custom-made MRI-compatible polyether ether ketone (PEEK) head holder and two bilateral ULTEM (an amorphous thermoplastic polyetherimide material) chambers (16 mm inner diameter) above the intraparietal sulcus (*ips*), embedded into surgical bone cement (Palacos, Zimmer BioMet), anchored to the cranium by ceramic screws (Rogue Research). The head holder and the chambers were designed by us and produced by the machine shop of the Physics Department at the California Institute of Technology. The California Institute of Technology Institutional Animal Care and Use Committee approved all surgical and animal procedures, which were performed in accordance with the National Institutes of Health Guide for the Care and Use of Laboratory Animals. Prior to the surgery, monkeys were trained to enter a vertical primate chair and perform basic reaching tasks. After recovery from the surgery, the animals were trained to maintain eye fixation and perform memory-guided saccade and reach tasks.

### Pharmacological inactivation

Microinfusions of the GABA-A agonist muscimol (Tocris Bioscience, MO) were made for each inactivation via a sterile 30-gauge stainless steel beveled-tip cannula (Plastics One). The cannula was affixed to a custom holder for the XYZ microdrive (FHC, Inc) and was lowered to the injection site through the custom-made ULTEM chamber grid inserts with 0.45 mm hole diameter and 0.8 mm inter-hole spacing (produced by the machine shop of the Physics Department at the California Institute of Technology). The muscimol was dissolved in phosphate-buffered saline (PBS) and the solution (5 mg/ml, pH 7.0–7.3) was sterile filtered (Corning Inc., NY) prior to injection. Total injection volumes ranged from 5.0–5.5 μl and were delivered at a rate of 1.0 μl/min using a 100 μl gas-tight Hamilton syringe driven by a digital infusion pump (Harvard Apparatus, MA). Injections were performed while the animals were awake and sitting comfortably in a custom chair, with their heads stabilized via the head holder. In absence of any hand-specific training, animals G and H spontaneously and consistently used the left and the right hand, respectively, to control the joystick. For this reason, all the injections were carried out in the contralateral hemisphere: right LIP in animal G and the left LIP in animal H. Each session started about 15–20 min after finishing the injections and lasted up to 2 h. Inactivation and control sessions were conducted in an alternate manner with a minimum interval of 24 hours^62^.

### Structural MRI acquisition

To identify the site of the injections, anatomical MR images of the brain were acquired in a Siemens TIM TRIO 3T horizontal bore scanner. The animals were sedated with Ketamine (10 mg/Kg) and Dexdomitor (0.02 mg/kg) and were intubated and maintained on isoflurane during the scans. The scans were performed using a standard 12 channel Siemens head coil. Anatomical scans were acquired with an MPRAGE sequence using the following parameters: TR = 1800 ms, TE = 3.55 ms, FOV = 179, slice thickness: 0.7 mm, in-slice resolution: 0.5 mm.

### Behavioral tasks

#### Memory-guided reaches

We used the same experimental task described in^24^. The animals sat in a dark room approximately 30 cm from an LCD monitor in a primate chair. A 2-dimensional joystick was positioned in front of the sitting animals, with the handle at the level of their knees. Each trial started with two central fixation cues presented in the center of the screen. The animals had to fixate their eyes on the red diamond cue (1.5 cm side length) and acquire the green circular cue (7.5 cm diameter) by moving a square cursor (0.3 cm side length) controlled by the joystick (Fig. 1A). If the animals moved the cursor outside the green circular cue or broke eye fixation (i.e. shift their gaze outside a tolerance window of 7.5 cm, corresponding to 14^°^ of visual angle) the trial was aborted and reward was withheld. After 1 s, either a single green circular cue (*instructed reach trial*) or two simultaneously appearing green circular cues (*free-choice reach trial*) were presented for 380 ms, indicating the location of the target(s). Instructed and free-choice trials were randomly interleaved in each session. In the choice trials, the two cues were simultaneously presented in both hemifields, equidistantly from the central fixation cue and symmetrically around the vertical axis of the screen. The cues were randomly selected from 2 potential locations with 23^°^ eccentricity (one in the right and one in the left hemifield) for monkey H, and from 4 potential locations - two in the left and two in the right hemifield – with low (17^°^) and high (25^°^) eccentricity for monkey G. Following cue offset, the animals were trained to remember the location of the cue(s) while maintaining both eye and hand fixation for 2.4 ± 0.5 s (mean ± SD) for monkey G and 1.15 ± 0.18 s for monkey H. Once the green hand fixation cue was extinguished (“go signal”), the animals had to move the cursor either to the remembered location of a single target or to a chosen target, while maintaining central fixation, and hold the cursor at the target location for another 300 ms before receiving the reward. Once the cursor entered the selected target area, target(s) were illuminated and stayed on for the duration of the hold period (in correctly completed trials), or were extinguished as soon as the target hold was aborted. Animals received the same amount of liquid reward in both successfully completed instructed and choice trials, and were rewarded equally for selecting either target in the choice trials. Incorrect trials with eye fixation aborts or reach initiation prior to go-signal or incorrect reaches were aborted and not rewarded.

#### Memory-guided saccades

Similar to the reaching task, we used a memory-guided saccade task with the main difference being that the location of the target(s) was indicated by a red diamond cue (1.5 cm side length) (Fig. 1B). The animals selected the target(s) by making eye movements. Only the central fixation cue (also a red diamond) was presented in the “initial fixation” period (i.e., no hand fixation was required). The memory period was 1.15 ± 0.28 s (mean ± SD) for both animals. Once the central fixation cue disappeared (“go signal”), the animals had to perform a saccade to  the  remembered single target (instructed trials) or to the chosen target (free-choice trials). The gaze had to arrive within a tolerance window of 7.5 cm diameter around the target and stay there for another 300 ms to receive the reward. The large window diameter was chosen for compatibility with the reaching trials. Animals received the same amount of liquid reward as in the reaching trials.

#### Ipsilesional and contralesional hemifields

Throughout the paper, we use the term “ipsilesional” and “contralesional” to refer to the visual hemifield with respect to the inactivated (in the inactivation sessions) hemisphere. For monkey H, the contralesional and the ipsilesional sides are the right and the left hemifields (injections were made into the left LIP). For monkey G, the contralesional and the ipsilesional sides are the left and the right hemifields (injections were made into the right LIP) (Fig. 2).

### Experimental procedures

Monkey H performed 13 control and 9 inactivation sessions, whereas monkey G performed 11 control and 4 inactivation sessions. Each session involved both reach and saccade tasks ran in separate blocks of trials. Control and inactivation sessions always started with the reach trials, followed by the saccade trials. The animals repeated the reaching task after completion of the saccade task in the inactivation sessions to ensure that the muscimol did not lose any efficacy during the saccade trials. A second block of the reaching task was also performed after the saccade trials in 3 control sessions in both animals. We found no systematic differences between the first and the last block in the reaching task (i.e., no differences on reaching performance – correct vs. incorrect trials – and on the choice preference) both in control and inactivation sessions. For this reason, reaching trials from the first and last block were pooled together for the remaining analyses. Inactivation sessions were interleaved with control sessions with a minimum interval of 24 hours. Per session, monkey H performed 355 ± 25 (mean ± SEM) and 317 ± 14 trials in the control and inactivation saccade task, respectively. He also performed 268 ± 18 and 345 ± 26 trials per session in the control and inactivation reaching task. Similarly, monkey G performed 380 ± 30 and 310 ± 4 per session in the control and inactivation saccade task, and 262 ± 28 and 259 ± 13 trials per session in the control and inactivation reaching task. Similar to our previous study^24^, control sessions did not include saline injections to minimize the potential risks from repeated injections.

### Stimulus presentation, online behavioral control, and data acquisition

Visual stimuli were presented on a vertical LCD monitor placed in the fronto-vertical plane approximately 30 cm from the animals’ eyes. The eye position was recorded with a miniature infrared camera (60 Hz; Resonance Technology, Inc) using ViewPoint software (Arrington Research). Reaching movements were performed using a 2-dimensional joystick (Measurement Systems, Inc). Joystick and eye positions were monitored at the same frequency (62.5 Hz), and were also recorded simultaneously with the stimulus and timing information. The visual stimulus presentation, online monitoring of eye and cursor position, and reward delivery were controlled by custom Python software based on the PsychoPy toolbox^63^.

### Behavioral data analysis

We evaluated the performance of the animals by computing the proportion of correct choices to the contralesional and ipsilesional hemifield in the instructed trials. We also measured the amplitude of the movements to test whether reaches and/or saccades became hypometric and/or hypermetric after LIP inactivation. The amplitude of the reach and saccade was computed as the angular distance the cursor and the eye, respectively, traveled during the movement. We also explored whether drug injection influences the reaction time (RT) of the movements. RT was defined as the time at which the movement velocity first exceeded 5% of the peak velocity. Finally, we computed the proportion of choices to contralesional and ipsilesional hemifields in the free-choice trials to test whether LIP inactivation affected the choice behavior. Only correct reach and saccade trials were used to compute the proportion of choices. Originally, we performed the analysis separately for each single target in the instructed trials and each pair of targets in the free-choice trials. Preliminary results showed no systematic effect of LIP inactivation on particular target locations within a given hemifield. The lack of the target location effect is likely due to (1) the large diameter of the fixation tolerance target window in the saccade trials and the large diameter of the cues in the reach trials, which resulted in a significant overlap between the targets, and, more importantly (2) the suppression of a large portion of LIP containing neurons with predominantly contralateral receptive fields that together represent the entire hemifield^48,64,65^. For this reason, the rest of the analysis is focused only on the hemifield differences between contralesional and ipsilesional targets regardless of their actual locations within a hemifield. Unless otherwise specified, we quantified the effects of LIP inactivation on visuomotor- and choice-behavior separately in each animal by performing a mixed factorial ANOVA across sessions with *effector* (joystick vs. eye) as a within-subject (i.e. within-session) factor and *treatment* as a between-subject (i.e. between-session) factor (control vs. inactivation). For performance, movement amplitude and reaction time (latency) the ANOVA was calculated on individual session mean values and separately for the contralesional and the ipsilesional hemifield. These analyses were followed by post hoc two-tailed *t* tests for statistical comparisons separately for each effector. The significance level was set to p < 0.05.

### Data availability

The datasets generated and analyzed during the current study are available from the corresponding author on reasonable request.
